# Performance Characteristics of a New Generation 148-cm Axial Field-of-View uMI Panorama GS PET/CT System with Extended NEMA NU 2-2018 and EARL Standards

**DOI:** 10.2967/jnumed.124.267963

**Published:** 2024-12

**Authors:** Haiqiong Zhang, Chao Ren, Yu Liu, Xinchun Yan, Meixi Liu, Zhixin Hao, Haiqun Xing, Li Huo

**Affiliations:** Department of Nuclear Medicine, State Key Laboratory of Complex Severe and Rare Diseases, Beijing Key Laboratory of Molecular Targeted Diagnosis and Therapy in Nuclear Medicine, Peking Union Medical College Hospital, Chinese Academy of Medical Sciences, Beijing, China

**Keywords:** NEMA, EARL, uMI Panorama GS PET/CT, LAFOV, time of flight

## Abstract

The uMI Panorama GS PET/CT system is a new long-axial-field-of-view scanner featuring high sensitivity, time-of-flight (TOF) resolution, spatial resolution, and count rate performance. The aim of this study is to assess the PET system on the basis of the National Electrical Manufacturers Association (NEMA) NU 2-2018 and European Association of Nuclear Medicine Research Limited (EARL) standards. **Methods:** Spatial resolution, count rate performance, sensitivity, accuracy, image quality, TOF resolution, and coregistration accuracy were evaluated following the NEMA NU 2-2018 standard. Additional experiments included energy resolution, 200-cm-long line sources for sensitivity, a 175-cm-long scatter phantom for count rate and TOF resolution, as well as the compliance with the EARL guideline. Moreover, an ^18^F-FDG PET patient study was reconstructed with various frame durations. **Results:** The PET system achieved sub–3-mm transaxial and axial spatial resolutions at a 1-cm radial offset. The sensitivities with the 70-cm-long and 200-cm-long line sources were observed to be 176.3 and 90.8 kcps/MBq, respectively, at the center of the field of view. The noise-equivalent count rates (NECRs) of the 70-cm-long and 175-cm-long scatter phantoms were measured to be 3.35 Mcps at 57.57 kBq/mL and 2.24 Mcps at 33.27 kBq/mL, respectively. The TOF resolutions for both phantoms were approximately 189 ps at 5.3 kBq/mL and lower than 200 ps below the NECR peaks. The absolute count rate errors of all 34 acquisitions were less than 3% below the NECR peak for the 70-cm-long scatter phantom. With the standard NEMA image quality phantom experiment, the contrast recovery coefficient varied from 68.17% to 94.20% and the background variabilities were all below 2%. The maximum PET/CT coregistration error was 1.33 mm. Regarding EARL compliance, the gaussian filter of 5-mm full width at half maximum could produce acceptable images. The patient data demonstrate visually satisfactory image quality with short frames (less than 1 min). **Conclusion:** The uMI Panorama GS exhibits spatial resolution and TOF resolution similar to those of the uMI Panorama system (35-cm axial field of view), despite the extended axial field of view. The 148-cm axial coverage, sub–200-ps TOF resolution, high sensitivity, and count rate performances are expected to yield superior image quality and offer new opportunities for various clinical applications.

## Visual Abstract

**Figure:**
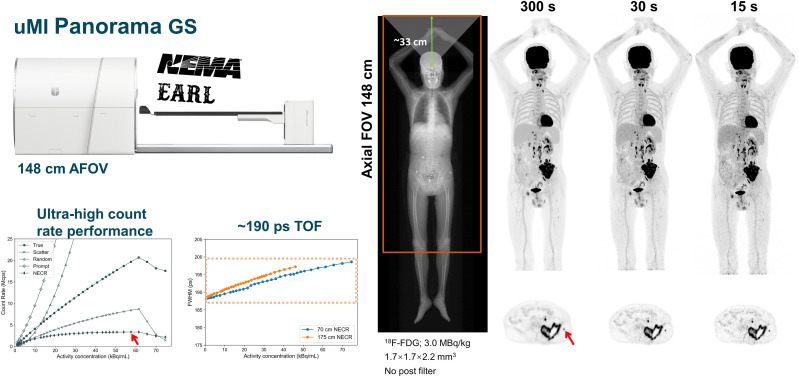

The integration of PET/CT systems, which was initially developed in the 1990s (1*,*^2^), has rapidly supplanted nearly all stand-alone PET systems. Over the past decade, it has emerged as a routinely used modality across various clinical applications.

Conventional clinical PET/CT systems typically encompass an axial field of view (AFOV) of less than 35 cm (3–9), whereby whole-body scans are achieved through multibed or continuous-bed-motion acquisitions (10). The limited sensitivity inherent in short AFOV scanners necessitates a trade-off between scan duration and image quality (IQ). Alternatively, the long-axial-field-of-view (LAFOV) scanners, characterized by their significant sensitivity gain, have brought novel insights into various molecular imaging applications (11*,*^12^), such as low-dose imaging (13*,*^14^), ultrafast scans (14), kinetic modeling (15*,*^16^), pediatric imaging (17*,*^18^), guided therapy (11), organ interaction research (11*,*^19^), and immunoPET imaging (20). Several LAFOV scanners, ranging from 64 to 194 cm, have been developed by several vendors and research entities, for example, the uEXPLORER (21), the PennPET Explorer (22*,*^23^), the Biograph Vision Quadra (24), and J-PET (25).

Aside from the evolution of LAFOV scanners, time-of-flight (TOF) resolution has dramatically improved from previous typical levels of approximately 500 ps to the current 200 ps (26). This notable advancement can be largely attributed to the use of fast scintillators and silicon photomultipliers in contemporary PET systems. The achieved TOF resolution has been demonstrated to enhance the IQ and lesion detectability or to reduce scan duration (27–30), thereby manifesting widespread application in clinical scans. Consequently, the advent of LAFOV scanners with improved TOF resolution emerges as a pivotal trend that is shaping the future landscape of PET imaging.

Among the presently developed LAFOV scanners, the PennPET Explorer stands as the pioneer, achieving in 2020 a TOF resolution of approximately 250 ps (22). Notably, its axial length has expanded from 64 cm (comprising 3 rings) (22) to 142 cm (comprising 6 rings) (23) over the past 4 y. However, the scanner remains in the prototype phase and has not yet been commercialized for wider clinical applications. The Biograph Vision Quadra, established in 2022, with a TOF resolution of approximately 220 ps and an axial length of 106 cm can provide coverage from head to thigh for people with average height (24). The system has been used for numerous clinical applications worldwide and has obtained improved IQ. Nonetheless, the line of response spanning the brain region is subject to a limited acceptance angle inherent to short AFOV scanners, due to its positioning at the axial periphery (31). Consequently, achieving a balance between IQ for both the brain and body within a single-bed scan, particularly for taller individuals, presents a considerable challenge. In contrast, the uEXPLORER, featuring an extended 2-m AFOV (21), offers the capability to position the subject’s head within a high-sensitivity axial region. However, the 505-ps TOF resolution (21) limits the achievable TOF gain.

In contrast, the recently established uMI Panorama platform (9) affords the flexibility to extend varying axial lengths, giving rise to the new LAFOV 148-cm-long scanner, denoted as the digital uMI Panorama GS PET/CT system. The 148-cm axial length of the PET scanner provides sufficient high sensitivity and adaptability for most clinical scans. The whole-body scan from head to thigh can position the brain within a considerably heightened sensitivity region. Alternatively, this scanner offers a head-to-knee scan option, and its axial length adequately accommodates nearly all pediatric imaging requirements. Total-body imaging is also feasible even for shorter subjects through knee bending, whereas taller patients exceeding 1.5 m benefit from the availability of 2-bed acquisitions.

This paper evaluates the PET system on the basis of the National Electrical Manufacturers Association (NEMA) NU 2-2018 standard (32), encompassing assessments of spatial resolution, sensitivity, scatter fraction, noise-equivalent count rate (NECR), accuracy, TOF resolution, IQ, and PET/CT coregistration accuracy. In addition, the IQ phantom with a sphere-to-background ratio of 10:1 underwent scanning and evaluation in accordance with the European Association of Nuclear Medicine Research Limited (EARL) guideline (33). Eventually, selected patient data were demonstrated to elucidate the clinical merits of this new LAFOV PET scanner.

## MATERIALS AND METHODS

### uMI Panorama GS PET/CT System

The uMI Panorama GS integrates a 160-slice CT scanner with a LAFOV PET system. The CT adheres to the identical configuration as the uMI Panorama (9). The specifications of PET system are given in Table 1. Benefiting from the extendable design of the uMI Panorama platform, the GS system combines 3 detector units, each equipped with 7 detector cells arranged along the axial direction (LYSO crystal array with full coverage of silicon photomultipliers), covering the AFOV of 148.2 cm.

**TABLE 1.** uMI Panorama GS System Specifications

Parameter	Specification
Crystal size	2.76 × 2.76 × 18.1 mm^3^
Microblock	3 × 3 crystal array coupled to 2 × 2 SiPM with pitch size of 2.85 mm
Detector cell	8 × 8 microblocks (24 × 24 crystal array)
Detector unit	7 cells along axial direction
	34 cells in transaxial direction
System	3 detector units
PET transaxial FOV	76.0 cm
PET AFOV	148.2 cm
Energy window	430–650 keV
Time window	4.7, 6.2, 8.3 ns for unit difference of 0, 1, 2
CT slices	160
Bore length with CT	2,263 mm
Maximal patient weight	250 kg
CT generator power	100 kW
CT minimal slice spacing	0.5 mm
Total system length	6,739 mm
System weight	6,100 kg

SiPM = silicon photomultiplier.

List-mode data are stored for clinical data acquisitions, with coincidences of all rings recorded (maximum acceptance angle of 62°). The time coincidence window is configured independently for different unit pairs to optimize coincidence retention within the field of view (FOV). The minimal discretized bin for TOF measurement is 6.1 ps.

### Measurements

Performance measurements in this paper were basically following the NEMA NU 2-2018 (32) and EARL standards (33). The acquired data for all experiments involved in this study were processed with the software tool kits provided by the manufacturer on the clinical system, including the nonstandard NEMA tests and the EARL study. The applied tool kits have undergone rigorous testing and validation by the manufacturer.

#### Spatial Resolution

^22^Na point sources (0.88 MBq, diameter of 0.25 mm) were applied for the spatial resolution measurement. Two axial planes at one-half AFOV and one-eighth AFOV were measured. For each axial plane, measurements were taken at 3 transaxial positions with radial offsets of 1, 10, and 20 cm, respectively. The acquired data were rebinned into 2-dimensional sinograms using the Fourier rebinning algorithm (34) with the oblique angle limited to 3.7°. Filtered backprojection reconstructions were performed without attenuation, scatter correction, or postfiltering, and the image voxel size was set to 0.6 × 0.6 × 0.8 mm^3^. The resolutions along the axial, radial, and tangential directions were calculated as instructed by the NEMA NU 2-2018 standard.

#### Sensitivity

Standard NEMA sensitivity measurements were performed using a 70-cm-long polyethylene tube filled with an initial activity of 8.9 MBq of ^18^F-FDG solution. The tube was added consecutively with 5 concentric aluminum sleeves of identical length and thickness (1.25 mm), each followed by a 5-min acquisition. Extrapolations of the 5 measured values were executed to obtain the final sensitivity. The procedure was repeated with the tube positioned at a 10-cm radial offset. Random subtractions were conducted using the coincidences within the delayed time window.

To evaluate the LAFOV scanner, additional experiments were conducted by applying a 2-m line source (10.5 MBq) along with a set of 2-m concentric aluminum sleeves following the same procedure as the aforementioned 70-cm-long sensitivity measurement.

#### Scatter Fraction, Count Losses, and Randoms Measurements

The phantom, also known as the NEMA scatter phantom, used for these measures is a 20-cm-diameter, 70-cm-long polyethylene cylinder positioned at the FOV center along the axial direction. A 70-cm-long tube was axially inserted into the cylinder phantom at a radial offset of 4.5 cm, which was injected with 1.7 GBq of ^18^F-FDG at the beginning of the acquisition. In total, 34 independent raw data acquisitions were performed, and the activity at the beginning of last acquisition was 26.0 MBq. Random estimation was extracted from the coincidences estimated within the delayed time window. Count rates for different coincidences, as well as scatter fraction and NECR count rates, were subsequently determined in accordance with the NEMA NU 2-2018 standard.

Additionally, the same experiment was repeated using a 175-cm-long phantom (21), formed by concatenating 2.5 70-cm scatter phantoms, with a penetrating tube. The tube was injected with 2.6 GBq of ^18^F-FDG at the beginning of the acquisition, and the activity at the beginning of the last acquisition was 40.1 MBq. Count rates, scatter fraction, and NECR count rates were analyzed following the NEMA NU 2-2018 standard across 34 acquisitions.

#### Accuracy: Corrections for Count Losses and Randoms

The accuracy analysis was performed using data obtained from the 70-cm-long NEMA scatter phantom assessing the count rate performances. The 34 acquisitions were reconstructed independently with all necessary corrections except for the decay correction. Dead time correction was applied using the adaptive nonparalyzable model (35). The reconstructions followed the current clinical protocol for GS at Peking Union Medical College Hospital (PUMCH) as listed here: ordered-subset expectation maximization/TOF/point-spread function, 3 iterations with 10 subsets, voxel size of 1.7 × 1.7 × 2.2 mm^3^, no postsmoothing. The accuracy analysis focused solely on the 65-cm center region along the axial direction, excluding 198 slices at each edge.

#### TOF Resolution and Energy Resolution

Data measured with 70-cm-long and 175-cm-long NEMA scatter phantoms were also evaluated for the TOF resolution (21). The line-source position within the scatter phantom was determined by reconstructing the image with non-TOF point-spread function reconstructions. All 34 acquisitions were parsed and analyzed in accordance with the NEMA NU 2-2018 standard by correcting for scatter, random, and line-source position. The TOF resolution at 5.3 kBq/mL was interpolated from the TOF–activity curves.

In addition to the TOF resolution, the energy spectra (4*,*^6^*,*^9^*,*^23^*,*^24^*,*^36^) were extracted from the 70-cm-long scatter phantom data, and the energy resolution of the full width at half maximum was determined using the same method used for the TOF resolution. The detailed method is described as in (9).

#### IQ and Accuracy of Corrections

The NEMA NU 2 IQ phantom was applied for this evaluation. The background activity was 5.0 kBq/mL at the beginning of the acquisition. A scatter phantom, also applied for the count rate measurement, was positioned adjacent to the IQ phantom. The line source inside the scatter phantom was filled with 105 MBq of ^18^F-FDG. Six spheres inside the phantom (with inner diameters of 10, 13, 17, 22, 28, and 37 mm) were filled with an activity ratio of 4.0:1 with respect to the background. The phantom was initially measured for 30 min. The data were reconstructed using the current clinical protocol for GS at PUMCH, incorporating all physical corrections, including decay, scatter, random, attenuation, normalization, and dead time. The contrast recovery coefficient (CRC) of all 6 spheres, the background variability (BV), as well as the lung residual were calculated as described in the NEMA NU 2-2018 standard.

To characterize the performance of the system with varying scan durations and delayed times (21), 4 additional acquisitions were conducted after 3, 6, 9, and 12 h, each measured for 30 min. Time frames of 0.5, 1, 2, 5, 10, and 30 min for each data were independently reconstructed. The CRC, BV, and lung residual were calculated for each reconstructed image. Note that for the time frames below 5 min, the CRC and BV were averaged over the results of 5 independent time frames.

#### PET/CT Coregistration Accuracy

Three spheres with an inner diameter of 13 mm, each filled with 2 MBq of ^18^F-FDG activity and CT contrast (Ultravist 370; Bayer Vital) mixture, were used to measure the coregistration accuracy of the PET and CT images according to the NEMA NU 2-2018 standard. Three spheres at nominal locations of (0, 1) cm, (0, 20) cm, and (20, 0) cm were fixed by the support pad (made of ethylene vinyl acetate foam, ∼900 Hounsfield units in CT images) at locations of 5 and 100 cm from the tip of the patient table. Meanwhile, lead blocks weighing 115 kg were placed on the patient table according to standard requirements. Both CT and PET images are reconstructed using the 1024 × 1024 matrix with a 600-mm FOV and a 0.5-mm slice thickness. Note that the PET images were reconstructed with an ordered-subset expectation maximization/TOF/point-spread function, 10 iterations and 3 subsets, without scatter and attenuation corrections. The maximum coregistration error and the maximum ratios of PET and CT were calculated following the NEMA NU 2–2018 standard.

#### EARL Performance

In addition to the NEMA standards, the quantitative recovery of the uMI Panaroma GS system was also assessed using an independent NEMA/International Electrochemical Commission NU2 IQ phantom following the EARL guideline. The phantom was filled with ^18^F-FDG at a concentration of 2.80 kBq/mL for the background at the beginning of the acquisition. All 6 spheres were filled with activity at a sphere-to-background ratio of 10. The phantom was positioned identically to the NEMA NU 2-2018 standard IQ experiment. A single-bed PET scan was acquired for 5 min. The data were reconstructed using the clinical protocol at PUMCH, incorporating all physical corrections. To conform to the EARL guideline, volumes of interest were extracted for each sphere to calculate the recovery coefficient of maximum, minimum, and peak values in the reconstructed image. Postgaussian kernels with various full widths at half maximum were applied to smooth the reconstructed image, ensuring compliance with the EARL protocol for this system.

#### Patient Study

An oncologic patient (height, 165 cm; weight, 55 kg), diagnosed with metastatic renal cell carcinoma, underwent a scan of 85 min after administration of 168 MBq (4.5 mCi) of ^18^F-FDG. A single-bed position from vertex to knee was acquired for 5 min. Time frames of 300, 180, 120, 60, 30, and 15 s were reconstructed from the list-mode data using the current clinical protocol for GS at PUMCH. The human study had been approved by the Institutional Review Board of PUMCH, and the patient had signed an informed consent form. Brain images were reconstructed from the same dataset with a voxel size of 0.6 × 0.6 × 0.6 mm^3^ and a gaussian filter of 0.5-mm full width at half maximum.

Two additional patients were also reconstructed and shown in the supplemental material (supplemental materials are available at http://jnm.snmjournals.org). One patient (height, 155 cm; weight, 74 kg) was injected with only 29 MBq (0.79 mCi) of ^18^F-FDG and scanned for 20 min. The other patient (height, 171 cm; weight, 160 kg) had a larger body mass index and underwent a scan of 110 min after administration of 444 MBq (12.0 mCi) of ^18^F-FDG.

## RESULTS

### Spatial Resolution

The spatial resolution results at 6 different positions are shown in Table 2. A sub–3-mm resolution is achieved at the center of the transaxial FOV.

**TABLE 2.** Spatial Resolution of NEMA NU 2-2018

			Full width at half maximum (mm)			Full width at 10th maximum (mm)		
FOV	Axial position (cm)	Radial position (cm)	Radial	Tangential	Axial	Radial	Tangential	Axial
**1/8 AFOV**	18.5	1	2.76	2.96	2.64	4.93	5.45	5.20
	18.5	10	3.29	3.02	2.96	5.62	5.53	5.54
	18.5	20	4.67	3.50	3.14	7.75	6.24	5.90
**1/2 AFOV**	74.0	1	2.82	2.91	2.91	4.79	5.54	5.42
	74.0	10	3.32	2.99	2.95	5.64	5.43	5.61
	74.0	20	4.65	3.15	3.15	7.85	6.54	6.00
**Average value of 1/8 and 1/2 AFOV**		1	2.79	2.94	2.78	4.86	5.50	5.31
		10	3.31	3.01	2.96	5.63	5.48	5.58
		20	4.66	3.33	3.15	7.80	6.39	5.95

### Sensitivity

**FIGURE 1.**
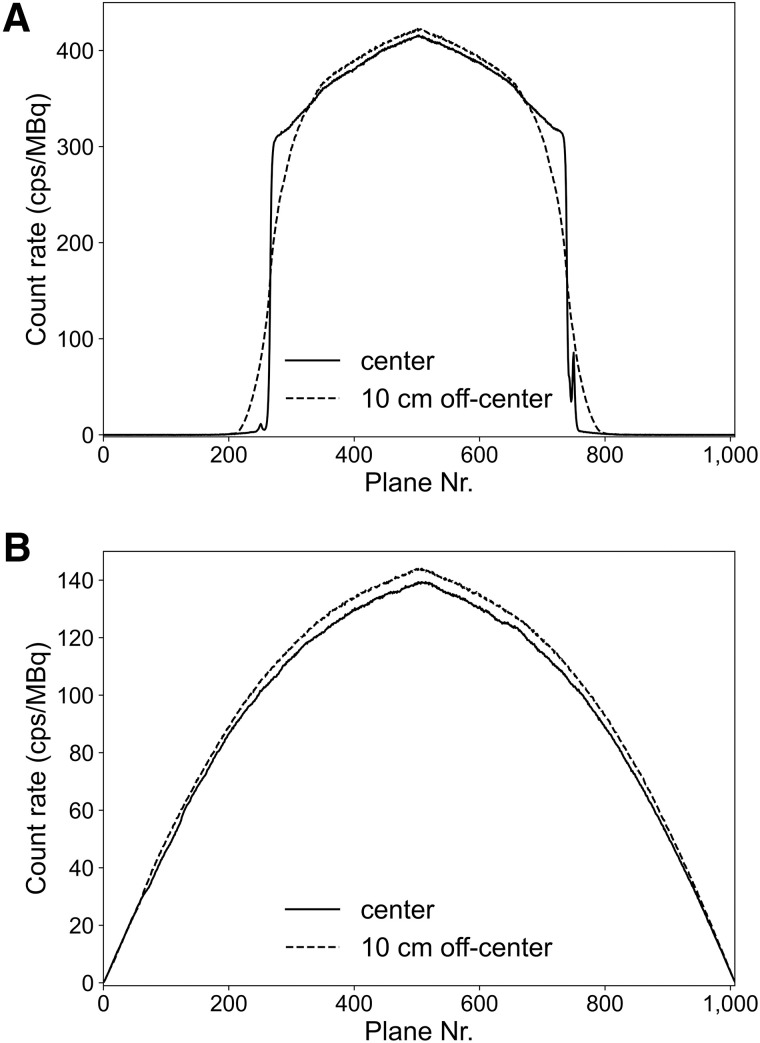
(A) Axial sensitivity profile of NEMA standard 70-cm-long line source positioned at transaxial center and 10 cm off-center. (B) Axial sensitivity profile of 200-cm-long line source positioned at transaxial center and 10 cm off-center. Nr. = number.

The sensitivity profiles of 70-cm-long and 200-cm-long line sources at the transaxial center and the off-center position (10 cm) are shown in Figure 1. The detailed system sensitivities are summarized in Table 3.

**TABLE 3.** Sensitivity Measured with 2 Line-Source Configurations

Line source	0 cm	10 cm
70 cm long	176.3 kcps/MBq	177.9 kcps/MBq
200 cm long	90.8 kcps/MBq	94.2 kcps/MBq

### Scatter Fraction, Count Losses, and Randoms Measurement

**FIGURE 2.**
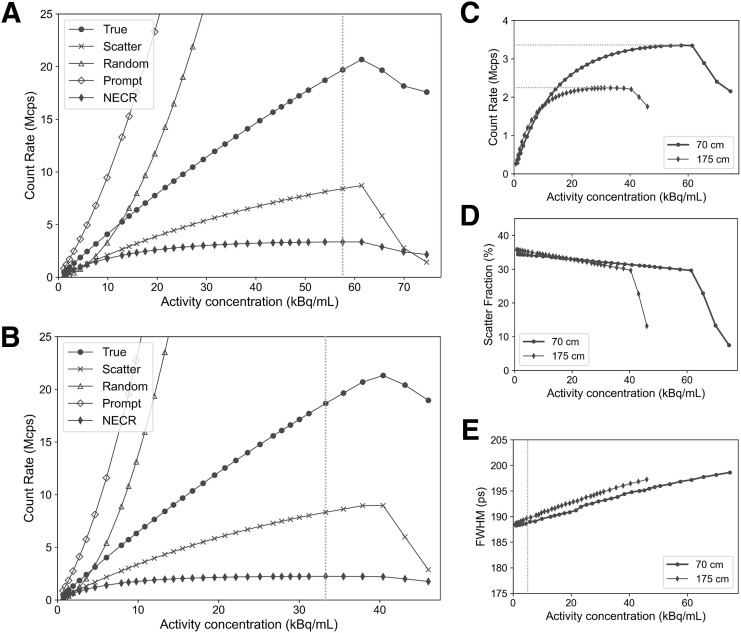
(A) Results for true, scatter, random, prompt, and NECR as functions of activity concentration for NEMA standard 70-cm scatter phantom. Dashed line corresponds to NECR peak. (B) Results for true, scatter, random, prompt, and NECR as function of activity concentration for 175-cm scatter phantom. Dashed line corresponds to NECR peak. (C) NECR as function of activity concentration for both 70-cm-long and 175-cm-long scatter phantoms. Dashed lines correspond to NECR peaks for both phantoms. (D) Scatter fraction as function of activity concentration for both 70-cm-long and 175-cm-long scatter phantoms. Scatter fractions at 5.3 kBq/mL for 70-cm-long and 175-cm-long phantoms are 34.13% and 35.09%, respectively, and scatter fractions at peak NECR are 29.94% and 30.85%. (E) TOF resolution as functions of activity concentration for both 70-cm-long and 175-cm-long scatter phantoms. Dashed line corresponds to 5.3 kBq/mL. TOF at 5.3 kBq/mL is 188.8 and 189.7 ps for 70-cm-long and 175-cm-long phantoms, respectively, and TOF at peak NECR is 196.8 and 195.2 ps. FWHM = full width at half maximum.

The profiles of the count rate performances for the 70-cm-long and 175-cm-long scatter phantoms are shown in Figure 2. Numeric results are summarized in Table 4. The peak NECRs of 70-cm-long and 175-cm-long scatter phantoms were measured to be 3.35 Mcps at 57.57 kBq/mL (total dose, 34 mCi) and 2.24 Mcps at 33.27 kBq/mL (total dose, 49.4 mCi), respectively. The 2 scatter phantoms demonstrate a similar scatter fraction (∼30%) at peak NECRs. The decrease of true activity after reaching the true peak is due to the system bandwidth limit, when the random count rate increases dramatically faster compared with that of true events.

**TABLE 4.** Count Rates, Scatter Fraction, and TOF Resolution

Parameter	70-cm-long phantom	175-cm-long phantom
Peak NECR	3.35 Mcps at 57.57 kBq/mL	2.24 Mcps at 33.27 kBq/mL
Peak true rate	20.68 Mcps at 61.41 kBq/mL	21.16 Mcps at 40.41 kBq/mL
Scatter fraction at peak NECR	29.94%	30.85%
TOF resolution at 5.3 kBq/mL	188.8 ps	189.7 ps

### Accuracy

**FIGURE 3.**
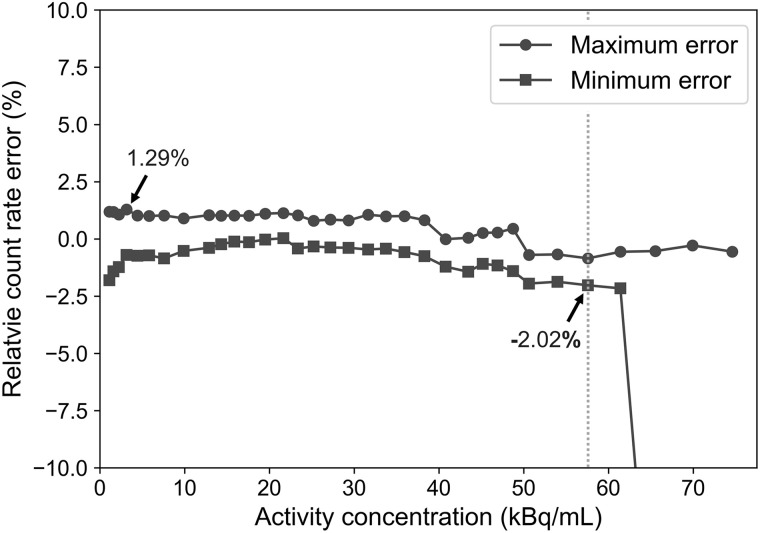
Accuracy of NEMA standard 70-cm-long scatter phantom. Dashed line corresponds to NECR peak. Turning points at high activity correspond to scenarios when measurement reached system bandwidth limit.

The accuracy of the standard 70-cm-long NEMA scatter phantom was calculated for the 34 acquisitions. The maximum and minimum errors of each slice among all the reconstructed images are visualized with respect to the activity in Figure 3. The absolute maximum and minimum count rate errors below the peak NECRs were observed to be 1.29% and 2.02%, respectively.

### Timing Resolution and Energy Resolution

The profile of TOF resolution is illustrated in Figure 2D, and the numeric results are presented in Table 4. For the applied scatter phantoms, the observed TOF resolution ranged from 188 to 197 ps across different activities below the NECR peaks. In addition, the measured energy resolution at 5.3 kBq/mL for the 70-cm-long NECR phantom was 9.7%.

### IQ and Accuracy of Corrections

The results for time frames of 30, 5, and 2 min are presented in Table 5. With the standard NEMA acquisition time (30 min), the CRC ranged from 68.17% to 94.20% and BV varied from 0.91% to 1.70% for the 6 hot spheres. Reconstructions of reduced time frames yielded higher BV for all spheres. In contrast, the CRC of large spheres fluctuated around the mean value with reduced time frames, whereas that of the 10-mm sphere decreased from 68.17% to 65.60%.

**TABLE 5.** CRC and BV Measurements

	30 min (NEMA)		5 min (clinical)		2 min (clinical)	
Sphere diameter	CRC (%)	BV (%)	CRC (%)	BV (%)	CRC (%)	BV (%)
10 mm	68.17	1.70	67.11	4.00	65.60	6.19
13 mm	76.09	1.48	76.40	3.30	77.94	5.02
17 mm	82.59	1.30	83.08	2.64	81.58	4.04
22 mm	86.79	1.11	86.36	2.10	86.79	3.24
28 mm	89.76	0.97	90.15	1.72	90.18	2.61
37 mm	94.20	0.91	93.79	1.40	94.20	2.07
Lung	1.87	—	1.98	—	1.98	—

**FIGURE 4.**
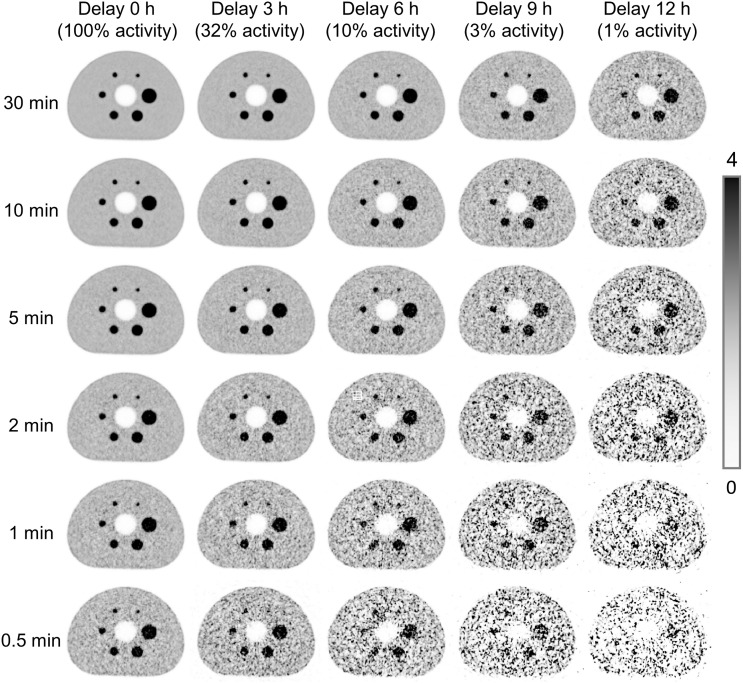
Reconstructed images at different delay times with various time frames.

**FIGURE 5.**
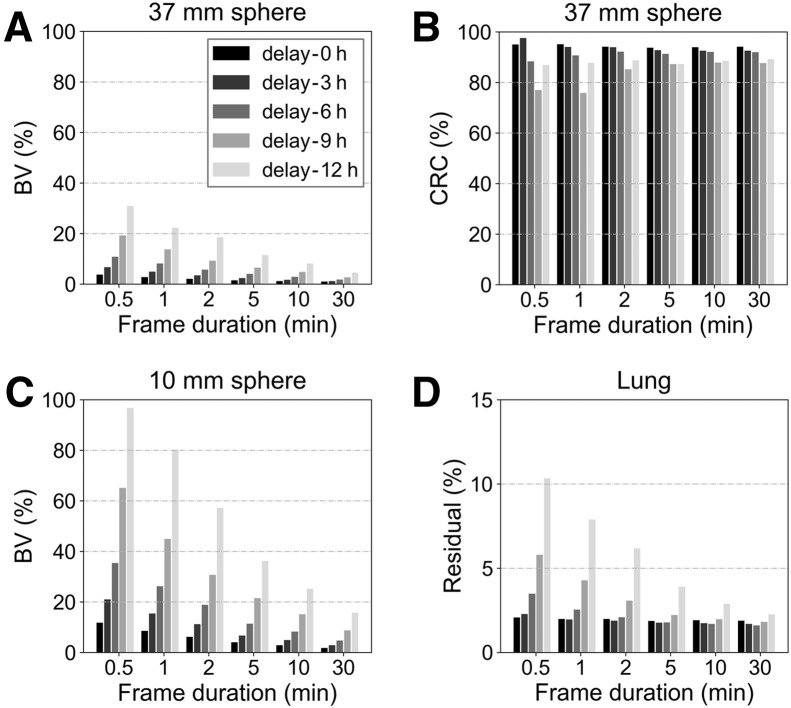
(A) BV of 37-mm sphere. (B) CRC of 37-mm sphere. (C) BV of 10-mm sphere. (D) Lung residual results.

The reconstructed images of different delay times and different time frames are demonstrated in Figure 4. The CRC and BV of the 37-mm sphere, the BV of the 10-mm sphere, as well as the lung residual error for all reconstructed images are demonstrated in Figure 5.

### PET/CT Coregistration Accuracy

The PET/CT coregistration accuracy results are shown in Table 6.

**TABLE 6.** PET/CT Coregistration Accuracy

Item	Measured Value
MaxCE	1.33 mm
R_max CT_	0.07
R_max PET_	0.11

MaxCE = maximum coregistration error; R_max CT_ = maximum ratio of CT; R_max PET_ = maximum ratio of PET.

### EARL Performance

**FIGURE 6.**
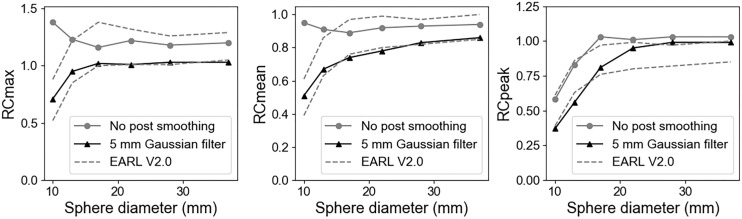
Recovery coefficients based on EARL guideline. Left to right: maximum recovery coefficient (RCmax) of all 6 spheres, mean recovery coefficient (RCmean) of all 6 spheres, and peak recovery coefficient (RCpeak) of all 6 spheres.

The recovery coefficients before and after smoothing are shown in Figure 6. Compared with the EARL version 2.0 standard region, most spheres exhibit relatively higher recovery coefficients, especially for the 10-mm and 13-mm spheres. With the gaussian filter of 5-mm full width at half maximum applied, nearly all spheres could fall into the EARL version 2.0 standard regions and satisfy this criterion.

### Patient Study

**FIGURE 7.**
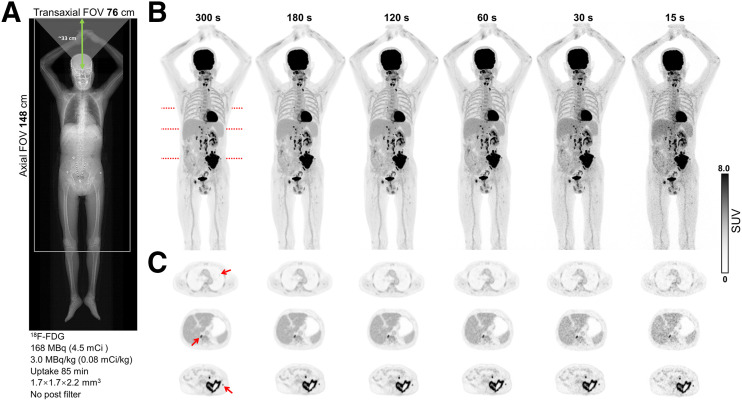
(A) Topogram, (B) maximum-intensity projection, and (C) transaxial images of patient data. Clear demarcations of reported lesions (indicated by arrows), including pulmonary nodule, retrocrural node, and subcutaneous nodule, are visible in each time frame.

The patient reconstructed images from various time frames are presented in Figure 7, revealing 3 representative lesions with subcentimeter diameters of 3.9, 15.7, and 7.4 mm discernible across all frames. The reconstruction of the 15-s frame demonstrated satisfactory lesion detectability. Noise contamination was evident at shorter time frames, whereas the 30-s frames maintained acceptable noise levels in the clinical images. In addition, Supplemental Figure 1 presents images of the brain with the same patient data. The low-dose and high–body mass index cases are shown in Supplemental Figures 2 and 3, respectively.

## DISCUSSION

The PET system has been assessed according to the NEMA NU 2-2018 standard (32) and the EARL guideline (33) along with some dedicated experiments to characterize LAFOV scanners.

In terms of spatial resolution, the measured results for the GS system are comparable to the reported results of uMI Panorama with a 35-cm AFOV, the entitled Panorama 35 in this paper. In fact, both systems are announced to be developed based on the same platform with identical transaxial geometry.

The sensitivity at the center position of the 70-cm-long line source achieved equivalent sensitivity (∼176 kcps/MBq) as the uEXPLORER (21) and Biograph Vision Quadra (24). Both the GS and uEXPLORER systems use identical crystal sizes and exhibit similar transaxial geometry. Despite the shorter AFOV compared with the uEXPLORER (acceptance angle of 57°), the results of GS are practically compensated by a larger acceptance angle (62°).

For LAFOV scanners with an AFOV beyond 70 cm, the NEMA standard line source becomes challenging in effectively demonstrating the actual sensitivity of a total-body scan (23). Longer line sources appear to be a theoretic necessity. The proposed experiment using a 200-cm-long line-source experiment offers scalability of results to various line-source configurations, as it penetrates the entire AFOV in contemporary PET systems. For instance, using a 170-cm-long line source, as applied in the uEXPLORER measurement (21), GS yields a scaled sensitivity of 106.8 kcps/MBq at the central transaxial FOV.

The NECR of the 70-cm-long phantom shows a lower peak activity at 57.57 kBq/mL compared with that of the Panorama 35 (9). Nevertheless, the current NECR and accuracy performances still cover a wide dynamic range for nearly all contemporary clinical applications (37).

The NECR of the 175-cm phantom is observed to be lower than that of the 70-cm-long phantom, mirroring the trend seen in the PennPET Explorer system with a 142-cm axial coverage (23). However, this trend differs from that observed in the uEXPLORER (21). On one hand, the current experiment configuration for GS holds a 27-cm-long phantom outside the AFOV, whereas the uEXPLORER can cover the entire phantom in a single bed. The radioactivity outside the AFOV contributes only to the scatter and random events but not to true events, theoretically resulting in reduced NECR performance. Additionally, the GS system has a larger coincidence window (ranging from 4.7 to 8.3 ns for different unit pairs) compared with that of uEXPLORER (ranging from 4.7 to 6.9 ns for different unit pairs) (21). The expanded time window is expected to capture more coincidences at the peripheral transaxial FOV. However, the increase in random events also occurs linearly with the extended time window, theoretically deteriorating the final numeric performance of NECR.

The peak true activities measured for the 70-cm-long and 175-cm-long scatter phantoms were 20.28 and 21.16 Mcps, respectively. Both measurements indeed reached the system bandwidth limit, as indicated by the turning points in the true activity curves. The scatter fraction performances for the 2 phantoms exhibit a consistent decreasing trend. These observations may be attributed to the energy peak shifting toward lower values, caused by the detector temperature rise at high count rates. Consequently, more scattered events were excluded by the low-level discriminator than by true events. In theory, the energy drift to lower values or increasing the low-level discriminator is considered to have a positive contribution to measured NECRs, as also reported (38) using a narrowed energy window.

The TOF resolution observed for the 70-cm-long scatter phantom data was less than 200 ps over the entire count rate range in the experiment, resembling the results of Panorama 35 (9). Similar TOF performances of these 2 systems demonstrate consistent detector designs of the Panorama platform, accurate system time synchronization, and appropriate time alignment. In terms of the 175-cm-long scatter phantom, the TOF resolution at 5.3 kBq/mL and peak NECRs are similar to those of the 70-cm-long scatter phantoms. These observations demonstrate stable TOF performances for different phantoms at various activity levels.

According to the NEMA NU 2-2018 standard, all spheres in the 30-min acquisition yield less than 1.7% BV, because of the proper physical corrections and high statistics acquired. The NEMA NU 2-2018 standard is not fully adapted to characterize the clinical performance for LAFOV scanners. Derived from the reconstructions of shorter time frames, the largest BV increases to 3.9% and 6.1% for 5 min and 2 min time frames, respectively. Despite this increase, the BV of the 5-min results still surpasses that of the Panorama 35, which was reconstructed with a longer time frame (6.8 min) and postsmoothing (9). Regarding the CRC, different reconstruction protocols make fair comparisons with the Panorama 35 or other scanners extremely difficult. Nevertheless, increasing CRCs with respect to the increasing of the sphere diameter is still observed for the GS system, exhibiting the same trend as the Panorama 35 and demonstrating tolerable Gibbs artifacts (39). Additionally, the CRC of small spheres, for example, 10 mm, showed lower CRCs with shorter time frames (Table 5), probably due to noise contamination and distortion of small structures with low statistics (40). This trend does not apply to larger spheres.

With a longer delay time of up to 12 h, the scanner is capable of producing visually acceptable images with 30-min acquisitions. These results are similar to those published for uEXPLORER (21) and can be explained by the comparable sensitivity between both scanners. A lung residual error of less than 3% was observed for all the reconstructed images of delayed scans at the NEMA standard acquisition time (30 min), which is largely due to the sub–200-ps TOF resolutions and proper physics corrections. Even under the extreme condition of a 0.5-min time frame with a delay time of 12 h, the lung residual error was still less than 10%. These performances demonstrate potential for ultra–low-dose applications, for example, immunoPET (20) or guided therapy (11).

For the image without postsmoothing, the biggest maximum recovery coefficient was observed for the 2 smallest spheres (diameters of 10 and 13 mm), which may be attributed to the small size of the volumes of interest and the resolution modeling. Similar results were reported for the Biograph Vision (36).

Based on the demonstrated patient images, clinical acquisitions below 1 min using the uMI Panorama GS can yield visually satisfactory IQ and sufficient lesion detectability for diagnostic purposes. The system’s high-sensitivity region (from the vertex to the thigh) and superior TOF resolution enable fast scans, proper dose reduction (as illustrated by the low-dose case in Supplemental Fig. 2), prolonged delayed imaging, and dynamic imaging of ultrashort frames. Concerning the neuroscience study, the brain center shown in Figure 7A is approximately 33 cm from the axial edge, resulting in an acceptance angle of approximately 41° and therefore yielding a sensitivity gain of ∼1.6 compared with that of the uMI Panorama 35 (acceptance angle of ∼25°), which can be visually supported by brain images shown in Supplemental Figure 1.

## CONCLUSION

The uMI Panorama GS demonstrates commensurate spatial resolution, TOF resolution, and energy resolution with the uMI Panorama system (35-cm AFOV). Nevertheless, its notably enhanced sensitivity yields improved IQ for the IQ phantom and patient. Achieving a peak NECR rate of 3.35 Mcps at 57.57 kBq/mL, alongside a less than 3% maximum bias, renders the system adaptable for a wide range of clinical and research applications. The combination of the above features positions the uMI Panorama GS as the new benchmark in the class of LAFOV scanners.

## DISCLOSURE

This study was sponsored in part by National High Level Hospital Clinical Research Funding (2022-PUMCH-B-071), CAMS innovation fund for medical science (Nos. CIFMS-2022-I2M-JB-001, CIFMS-2021-I2M-1-025, CIFMS-2021-I2M-1-002, CIFMS-2021-I2M-1-003), and National Key Research and Development Program of China (No. 2016YFC0901500). No other potential conflict of interest relevant to this article was reported.

## Notes

KEY POINTS **QUESTION:** What are the performance characteristics of the uMI Panorama GS PET/CT system, the new LAFOV scanner, according to the NEMA NU 2-2018 and EARL standards? **PERTINENT FINDINGS:** The uMI Panorama GS with a 148-cm AFOV demonstrated high sensitivity, temporal resolution (<200 ps), and spatial resolution (<3 mm at center), accompanied by a remarkable count rate performance and quantitative accuracy. Using appropriate filtering (5-mm gaussian), the system demonstrates capability to attain EARL compliance, thus revealing its harmonization capabilities with other scanners. **IMPLICATIONS FOR PATIENT CARE:** The uMI Panorama GS offers highly effective diagnostic capability on lesion detectability with reduced dose or acquisition time. Meanwhile, the system exhibits versatility in accommodating applications using various tracers, particularly in high count rate scenarios.
